# The Effects of Quassinoid-Rich *Eurycoma longifolia* Extract on Bone Turnover and Histomorphometry Indices in the Androgen-Deficient Osteoporosis Rat Model

**DOI:** 10.3390/nu10070799

**Published:** 2018-06-21

**Authors:** Putri Ayu Jayusman, Isa Naina Mohamed, Ekram Alias, Norazlina Mohamed, Ahmad Nazrun Shuid

**Affiliations:** 1 Department of Pharmacology, Faculty of Medicine, Universiti Kebangsaan Malaysia, Jalan Yaacob Latif, Cheras 56000, Kuala Lumpur, Malaysia; putri.ayujay@gmail.com (P.A.J.); isanaina@yahoo.co.uk (I.N.M.); azlina@ppukm.ukm.edu.my (N.M.); 2 Department of Biochemistry, Faculty of Medicine, Universiti Kebangsaan Malaysia, Jalan Yaacob Latif, Cheras 56000, Kuala Lumpur, Malaysia; ekram.alias@ppukm.ukm.edu.my

**Keywords:** *Eurycoma longifolia*, degarelix, testosterone, osteoporosis

## Abstract

Male osteoporosis is associated with higher rates of disability and mortality. Hence the search for suitable intervention and treatment to prevent the degeneration of skeletal health in men is necessary. *Eurycoma longifolia* (EL), a traditional plant with aphrodisiac potential may be used to treat and prevent male osteoporosis. The skeletal protective effect of quassinoid-rich EL extract, which has a high content of eurycomanone, has not been studied. This study aimed to determine whether EL could prevent skeletal deteriorations in gonadal hormone-deficient male rats. Ninety-six male Sprague–Dawley rats were randomly assigned to baseline, sham-operated (Sham), orchidectomised or chemically castrated groups. Chemical castration was achieved via subcutaneous injection of degarelix at 2 mg/kg. The orchidectomised and degarelix-castrated rats were then divided into negative control groups (ORX, DGX), testosterone-treated groups (intramuscular injection at 7 mg/kg weekly) (ORX + TES, DGX + TES), and EL-supplemented groups receiving daily oral gavages at doses of 25 mg/kg (ORX + EL25, DGX + EL25), 50 mg/kg (ORX + EL50, DGX + EL50), and 100 mg/kg (ORX + EL100, DGX + EL100). Following 10 weeks of treatment, the rats were euthanized and their blood and femora were collected. Bone biochemical markers, serum testosterone, osteoprotegerin (OPG), and receptor activator of nuclear factor kappa β-ligand (RANKL) levels and histomorphometric indices were evaluated. Quassinoid-rich EL supplementation was found to reduce degenerative changes of trabecular structure by improving bone volume, trabecular number, and separation. A reduction in the percentage of osteoclast and increase in percentage of osteoblast on bone surface were also seen with EL supplementation. Dynamic histomorphometric analysis showed that the single-labeled surface was significantly decreased while the double-labeled surface was significantly increased with EL supplementations. There was a marginal but significant increase in serum testosterone levels in the ORX + EL25, DGX + EL50, and DGX + EL100 groups compared to their negative control groups. Quassinoid-rich EL extract was effective in reducing skeletal deteriorations in the androgen-deficient osteoporosis rat model.

## 1. Introduction

Sex steroids play a crucial role in the development and maintenance of the skeletal system in human and in experimental animals [1]. Androgens modulate the bone remodeling cycle through direct androgenic activity via androgen receptors that are present on bone compartments or by indirect action through aromatization into estrogens [2]. It has been established that lack of estrogen in females causes rapid bone loss and lack of androgen in males induces osteopenia. Although osteoporosis is less common in men compared to women, it has been recognized that the morbidity and mortality after osteoporotic fractures in men are a major public health issue. The major causes of osteoporosis reported in men have been mainly separated into primary (age-related and idiopathic osteoporosis) and secondary causes (alcohol abuse, glucocorticoid excess, and hypogonadism) [3]. Hypogonadism (i.e., reduction in circulating androgens level) has been associated with low bone mineral density (BMD) and an increased risk of fractures [4].

The orchidectomised male rat has been widely used as an animal model for the study of male osteoporosis related to androgen deficiency [5]. Androgen deficiency in male rats caused a substantial loss of cancellous bone that was associated with a sustained increase in bone turnover [6]. Administration of pharmaceutical agents such as gonadotropin releasing hormone (GnRH) agonists, androgen receptor antagonists, and aromatase inhibitors were reported to cause osteopenia in experimental animals [7,8]. In adult men, androgen withdrawal by surgical or chemical castration induced high turnover bone loss [6]. Orchidectomy and hormonal therapy such as GnRH agonists buserelin, goserelin, and leuprolide are the androgen deprivation therapies available for prostate cancer patients [9]. These therapeutic options have been linked to secondary osteoporosis in men [10].

In addition to agonists, GnRH antagonists have been developed as a new approach for the treatment of prostate cancer [9]. Degarelix is a potent and long-acting GnRH antagonist that is capable of producing a rapid reduction of testosterone through their competitive binding to GnRH receptors [11]. Therefore, it is anticipated that degarelix administration may also cause profound bone loss due to hypothalamic-pituitary-gonadal (HPG) suppression. However, to date, the adverse skeletal changes following GnRH antagonist, degarelix administration have never been studied. This study utilized both surgical and chemical castration for development of androgen-deficient osteoporosis rat models. Administration of a potent GnRH antagonist, degarelix in intact rats and surgical castration with bilateral orchidectomy may cause testosterone-deficient osteoporosis in experimental rats.

Clinical trials have shown that long-term testosterone replacement therapy (TRT) moderately increased BMD in hypogonadal men with osteoporosis [12]. Combination of TRT and anti-resorptive therapy such as alendronate to hypogonadal men produced more pronounced effects [13]. However, these pharmacological agents are not free from side effects [14,15]. For instance, TRT has been associated with increased risks of stroke and cardiovascular events due to the increase in blood viscosity [16]. TRT is also not recommended for patients with underlying or high-risk factors of cancer [17]. Upper gastro-intestinal tract complaints were reported in osteoporotic patients with history of taking alendronate medication [18]. Long-term use of alendronate in the treatment of osteoporosis must be performed with more caution as it may result in a new form of insufficiency fracture of the femur due to prolonged suppression of bone remodeling [19]. Hence the search for a natural alternative with similar efficacy but free from side effects is highly desirable.

*Eurycoma longifolia* (EL) or locally known as Tongkat Ali in Malaysia, is an evergreen slow-growing herbal plant that is classified under the *Simaroubaceaea* family [20]. Almost all parts of this plant, especially the roots have been used by traditional practitioners to treat various ailments including sexual insufficiencies, dysentery, persistent fever, and malaria [21]. The roots of EL contain a wide variety of bioactive compounds including alkaloids, quassinoids, quassinoid diterpenoids, eurycomaoside, eurycolactone, laurycolactone or eurycomalactona which are responsible for most of the reported health benefits [20]. Numerous animals studies have been carried out supporting the aphrodisiac and testosterone enhancing potentials of EL root extract [22,23]. Based on clinical evidence, the roots of EL extract showed the potential to improve sexual problems such as erectile dysfunction and appeared to be useful for the management of hypogonadism [24,25].

It was shown that EL was capable of restoring serum testosterone level, thus significantly improving sexual health [26]. Due to its androgenic properties, EL may have the potential to treat diseases related to androgen deficiency including bone loss. The root extract of this plant was reported to possess anti-osteoporotic activities. Earlier study demonstrated that the aqueous EL extract was able to prevent bone calcium loss [27]. The combination of EL extract and low-dose testosterone treatment for six weeks was also able to reduce bone turnover in orchidectomised rats [28]. However, in another study, supplementation of EL extract alone failed to emulate testosterone action in reducing degenerative changes on bone volume due to orchidectomy-induced testosterone deficiency [29].

The aqueous extract of EL that was used in previous anti-osteoporotic studies contained 22.0% eurypeptide, 41.1% glycosaponin, and 1.6% eurycomanone. Earlier studies suggested that glycopeptide in the aqueous extract of EL was responsible for its aphrodisiac and fertility-enhancing effects [25,30]. However, the methanol extract of EL, which did not contain any glycopeptide also displayed similar potential [31]. Both aqueous and methanol extracts of EL were reported to have quassinoid amounts including eurycomanone but at lower concentrations. Hence, it was postulated that the quassinoid, eurycomanone, which is indigenous in *Simaroubaceaea* plants, was responsible for the reported effects. More recently, quassinoid-rich EL extracts that contain more than 10-fold of eurycomanone and its analogue were studied for its androgenic potential [32,33]. It was found that quassinoid-rich EL extracts might be worthy of further development as phytomedicine to treat male disease related to testosterone deficiency.

The present study was therefore undertaken to examine the effects of quassinoid-rich EL extracts at 25, 50, and 100 mg/kg body weight for 10 weeks on bone turnover and histomorphometry indices of androgen-deficient rats. To determine the effects of quassinoid-rich EL extract on bone remodeling, osteocalcin, a bone formation marker, and C-terminal telopeptide of type 1 collagen (CTX), a bone resorption marker, were measured. The structural, static and dynamic histomorphometric parameters were evaluated to provide a useful profile of bone turnover [34]. The testosterone-raising ability of quassinoid-rich EL extract was thought to be responsible for its protective effects on bone. In the present study, the androgenic potential of quassinoid-rich EL extract was confirmed by the measurement of serum testosterone level. As androgen also appeared to regulate the levels of osteoprotegerin (OPG) and receptor activator of nuclear factor kappa β-ligand (RANKL) [35,36] and modification of the RANKL-OPG signaling pathway has major effects on bone remodeling [37], serum OPG and RANKL levels were measured in the androgen-deficient rat model.

## 2. Materials and Methods

### 2.1. Animals and Treatment

The study was conducted in accordance with the recommendations of Universiti Kebangsaan Malaysia (UKM) Animal Ethics Committee (Approval Code: FP/FAR/2015/NAZRUN/25-MAR./665-MAR.-2015-DEC.-2017). Ninety-six intact-male Sprague–Dawley rats (3 months old) were allowed to acclimatize to an environmentally controlled room (12-h light/dark cycle, room temperature) in an animal care facility for one week. The animals were housed in plastic cages and were given access to standard pellet diet and water ad-libitum.

Then, the rats (weighing between 300 g and 350 g) were randomized into four main groups, which were the baseline group (Baseline, *n* = 8), sham-operated (Sham, *n* = 8), surgically- and chemically-castrated groups. The baseline rats that did not undergo any surgical treatment or intervention were sacrificed at the beginning of the study. Surgical castration was carried out by orchidectomy, where both testes were permanently removed under anesthesia. Chemical castration was performed via two subcutaneous injections of degarelix at a dose of 2 mg/kg with the testes remaining intact. The first injection was given at the beginning of the study and the second injection 6 weeks later. The surgically castrated rats which consisted of forty rats were further subdivided into five groups of orchidectomised control (ORX), orchidectomised and given testosterone at 7 mg/kg (ORX + TES), orchidectomised and given standardized quassinoid-rich EL extracts at 25 mg/kg (ORX + EL25), 50 mg/kg (ORX + EL50) and 100 mg/kg (ORX + EL100). The chemically castrated groups were subdivided into degarelix-induced control (DGX), degarelix-induced, and given testosterone at 7 mg/kg (DGX + TES), degarelix-induced and given standardized quassinoid-rich EL extracts at 25 mg/kg (DGX + EL25), 50 mg/kg (DGX + EL50), and 100 mg/kg (DGX + EL100) with eight rats in each group. One week of recovery period from castration procedures was given to the rats before starting the treatments. Testosterone enanthate was injected intramuscularly once a week while the standardized quassinoid-rich EL extracts were given daily for 10 weeks via oral gavages.

### 2.2. Blood and Bone Sampling

Rats in the baseline group were killed by ether overdose before the start of the study while the other rats were sacrificed upon completion of their treatments. The rat bones were fluorochrome-labelled by intraperitoneal injection of 20 mg/kg calcein for dynamic histomorphometry measurements at 9 days and 2 days before euthanasia. The rats were fasted overnight before blood collection was performed. Blood was collected from the orbital sinus. After three hours at room temperature, serum was extracted by centrifugation (3000 rpm × 10 min) and stored at −80 °C until biochemical analyses were performed. The left femora were dissected out and prepared for histomorphometric analyses.

### 2.3. Bone Biochemical Markers

Serum osteocalcin and C-terminal telopeptide of type 1 collagen (CTX) were measured using an enzyme-linked immunosorbent assay (ELISA) technique and analyzed using ELISA reader (VERSAmax, Sunnyvale, CA, USA). The kits used were rat osteocalcin ELISA (IDS, Tyne & Wear, Boldon Colliery, UK) and Ratlaps ELISA CTX (IDS, Nordic Biosciences, Boldon Colliery, UK).

### 2.4. Bone Histomorphometry

The histomorphometric parameters were defined as structural, static, and dynamic indices according to the American Society for Bone and Mineral Research Committee. The distal part of the left femora was sawed into halves and fixed with 10% neutral-buffered formalin. One part of the bone samples was decalcified using ethylenediaminetetra-acetic acid (Sigma-Aldrich, St. Louis, MO, USA) while the other part was processed as undecalcified specimens. Decalcification was carried out for 8 weeks during which time the decalcifying solution was changed weekly. The decalcified bones were dehydrated and processed to form paraffin blocks. Paraffin sections of 5.0 μm thick were stained with hematoxylin and eosin stain (H & E).

The undecalcified specimens were embedded in polymer methyl methacrylate (Osteo-Bed Bone Embedding Kit; Polysciences, Warrington, PA, USA) and sectioned at 9.0 μm thickness using a microtome (Leica RM2155, Wetzlar, Germany). The sections were then stained using Von Kossa’s method for structural histomorphometry. The histological slides were analyzed using Nikon Eclipse 80i microscope (Nikon Instrument Inc., Melville, NY, USA) with an image analyzer software Pro-Plus v. 5.0 (Media Cybernatics, Silver Spring, MD, USA) and a Weibel grid as described previously (Parfitt et al. 1987). The unstained bones were analyzed for dynamic bone parameters using a fluorescence microscope (Nikon Eclipse 80 μ, Nikon, Tokyo, Japan) and an image analyzer Pro-Plus (Media Cybernetics, Silver Spring, MD, USA). The measurements were performed at the metaphyseal region, which is located 1 mm from growth plate, excluding the endocortical region. This is the secondary spongiosa area, which is rich in trabecular bone. Trabecular bone was chosen because its remodeling process is more dynamic than the cortical bone. All the histomorphometric parameters measured were listed in Table 1.

### 2.5. Serum Testosterone, OPG & RANKL

Testosterone was measured in blood using a mouse/rat testosterone ELISA kit (BioVendor, Brno, Czech Republic), with a low detection limit of 0.066 ng/mL. Serum level of OPG and RANKL were measured with commercially available ELISA kit (Elabscience Biotechnology Co., Ltd., Wuhan, China) and detected with a multimode microplate reader (EnSpire Multimode, Perkin Elmer, Singapore).

### 2.6. Statistical Methods

Results were expressed as mean ± standard error of the mean (SEM). All data were analyzed using the Statistical Package for the Social Sciences (SPSS, version 23.0, Chicago, IL, USA). Distribution of the data was assessed using the Shapiro–Wilk test. A parametric one-way analysis of variance (ANOVA) was performed to detect any significant difference among the groups. If the results were significant (*p* ≤ 0.05), the Multiple Comparisons Post Hoc Tests, either Tukey’s or Dunnett’s T3 were used to determine the specific differences between means: *p*-value less than 0.05 was considered as statistically significant.

## 3. Results

### 3.1. Body Weight

**Figure 1:**
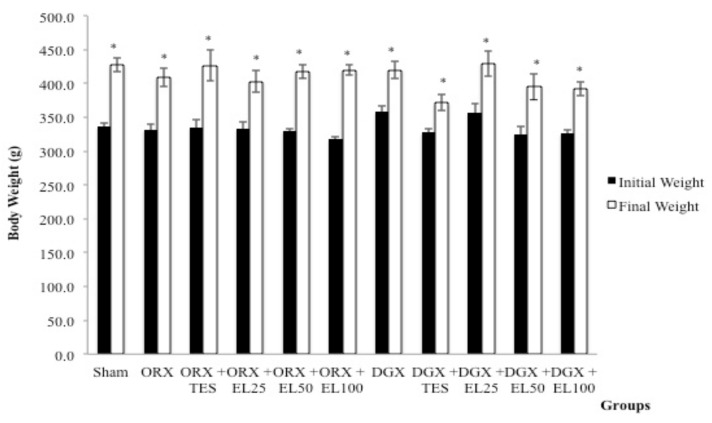
Mean body weight before and after treatment. Data expressed as mean ± standard error of the mean (SEM.) *n* = 8 rats in each group. *p* < 0.05 is considered as significant. Abbreviations: orchidectomised = ORX; TES = testosterone; DGX = degarelix-induced; EL = *Eurycoma longifolia*. Experimental groups: Sham, Sham-operated; ORX, orchidectomised control; ORX + TES, orchidectomised + testosterone injection; ORX + EL25, orchidectomised + 25 mg/kg EL extract; ORX + EL50, orchidectomised + 50 mg/kg EL extract; ORX + EL100, orchidectomised + 100 mg/kg EL extract; DGX, degarelix-induced; DGX + TES, degarelix-induced + testosterone injection; DGX + EL25, degarelix-induced + 25 mg/kg EL extract; DGX + EL50, degarelix-induced + 50 mg/kg EL extract; DGX + EL100, degarelix-induced + 100 mg/kg EL extract * Significant difference versus baseline of the same group.

At the beginning of the study, the difference in the mean body weight for all groups was not statistically significant (*p* > 0.05). Following 10 weeks of treatment, the mean body weight within groups was significantly increased but there was no significant difference (*p* > 0.05) between groups (Figure 1).

### 3.2. Bone Biochemical Markers

**Figure 2:**
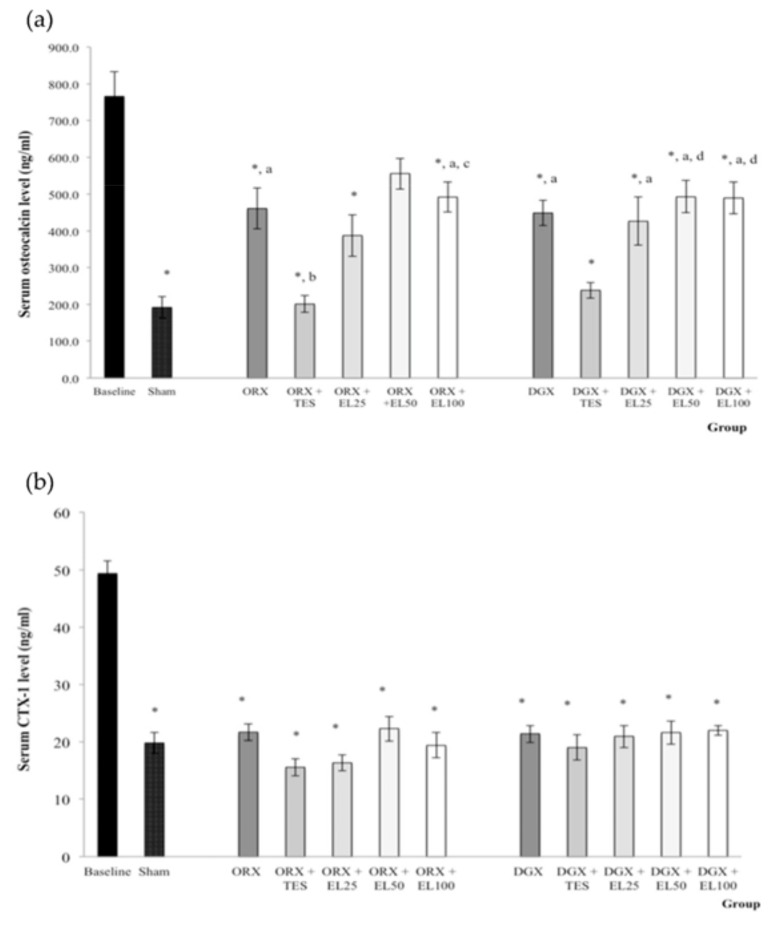
(**a**) Mean osteocalcin level in the serum after treatment; (**b**) Mean C-terminal telopeptide of type 1 collagen (CTX-1) level in the serum after treatment. Data expressed as mean ± SEM. *n* = 8 rats in each group. *p* < 0.05 is considered as significant. Abbreviations: orchidectomised = ORX; TES = testosterone; DGX = degarelix-induced; EL = *Eurycoma longifolia*. Experimental groups: Sham, Sham-operated; ORX, orchidectomised control; ORX + TES, orchidectomised + testosterone injection; ORX + EL25, orchidectomised + 25 mg/kg EL extract; ORX + EL50, orchidectomised + 50 mg/kg EL extract; ORX + EL100, orchidectomised + 100 mg/kg EL extract; DGX, degarelix-induced; DGX + TES, degarelix-induced + testosterone injection; DGX + EL25, degarelix-induced + 25 mg/kg EL extract; DGX + EL50, degarelix-induced + 50 mg/kg EL extract; DGX + EL100, degarelix-induced + 100 mg/kg EL extract * Significant difference versus baseline; ^a^ Significant difference versus Sham; ^b^ Significant difference versus ORX; ^c^ Significant difference versus ORX + TES; ^d^ Significant difference versus DGX.

The serum osteocalcin level of all groups was significantly lower than the baseline group except for ORX + EL50 group. The serum osteocalcin level of ORX + EL100, DGX, DGX + EL25, DGX + EL50, and DGX + EL100 groups were significantly higher than Sham group (*p* < 0.01). The serum osteocalcin level of ORX + TES group was significantly lower than ORX and ORX + EL100 groups (*p* < 0.05). The serum osteocalcin level of DGX + TES group was also lower than DGX, DGX + EL50 and DGX + EL100 groups but the differences were only significant for the latter two groups (*p* < 0.05) (Figure 2a).

The CTX-1 levels were significantly lower in all groups compared to the baseline group (*p* < 0.001). There were no other significant findings (Figure 2b).

### 3.3. Serum OPG and RANKL Level

**Figure 3:**
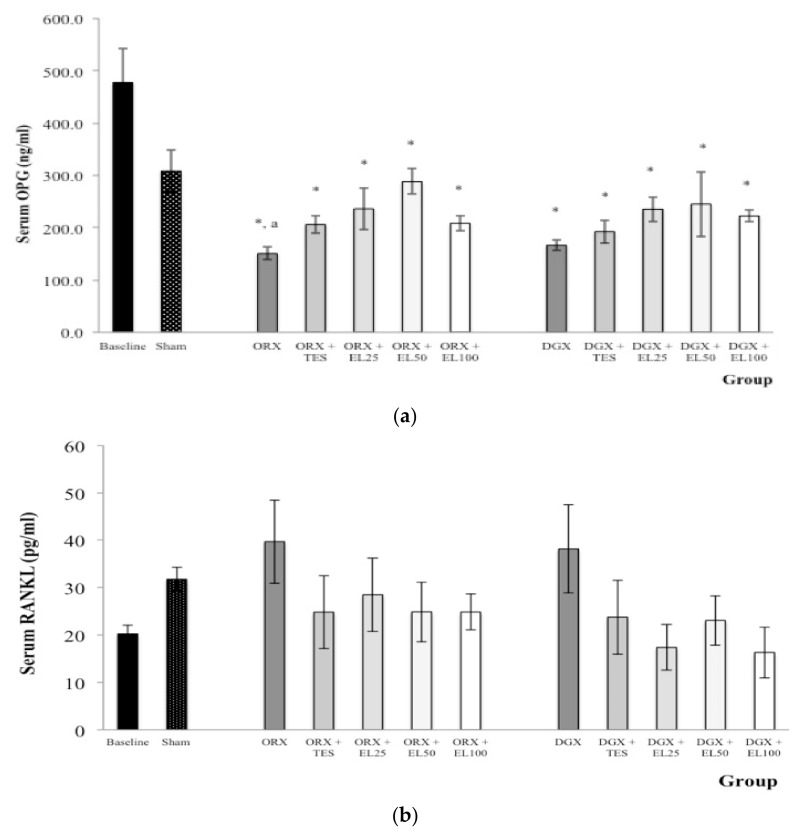
(**a**) Mean osteoprotegerin (OPG) level in the serum after treatment; (**b**) Mean receptor activator of nuclear factor kappa β-ligand (RANKL) level in the serum after treatment. Data expressed as mean ± SEM. *n* = 8 rats in each group. *p* < 0.05 is considered as significant. Abbreviations: orchidectomised = ORX; TES = testosterone; DGX = degarelix-induced; EL = *Eurycoma longifolia*. Experimental groups: Sham, Sham-operated; ORX, orchidectomised control; ORX + TES, orchidectomised + testosterone injection; ORX + EL25, orchidectomised + 25 mg/kg EL extract; ORX + EL50, orchidectomised + 50 mg/kg EL extract; ORX + EL100, orchidectomised + 100 mg/kg EL extract; DGX, degarelix-induced; DGX + TES, degarelix-induced + testosterone injection; DGX + EL25, degarelix-induced + 25 mg/kg EL extract; DGX + EL50, degarelix-induced + 50 mg/kg EL extract; DGX + EL100, degarelix-induced + 100 mg/kg EL extract * Significant difference versus baseline; ^a^ Significant difference versus Sham.

Orchidectomy and DGX administration resulted in significantly lower serum OPG in all orchidectomised and degarelix-induced groups compared to the baseline group (*p* < 0.05). The reduction in serum OPG was only significant in the ORX group when compared to the Sham group (*p* < 0.05) (Figure 3a). There were no significant differences in serum RANKL for all experimental groups (*p* > 0.05) (Figure 3b).

### 3.4. Serum Testosterone Level

The serum testosterone level was significantly higher in ORX + TES and DGX + TES groups but was found to be significantly lower in all other groups compared to the baseline group (*p* < 0.001) (Table 2). There were significant reductions in serum testosterone levels for all orchidectomised and degarelix-induced groups compared to the Sham group (*p* < 0.05), except for ORX + TES and DGX + TES groups, which were significantly higher than the Sham group and their respective negative control groups (*p* < 0.001). The serum testosterone level in ORX + TES and DGX + TES groups was also significantly higher compared to the EL-supplemented groups (*p* < 0.05). The marginal increase in the serum testosterone level in ORX + EL25, DGX + EL50 and DGX + EL100 groups was significant when compared to their negative control groups (*p* < 0.01).

### 3.5. Static Histomorphometric Parameters

ObS/BS for ORX + EL50, DGX + TES, DGX + EL25, DGX + EL50 and DGX + EL100 groups was significantly higher than the baseline group (*p* < 0.01). The ObS/BS of DGX+TES, DGX + EL25 groups was also significantly higher than the Sham and DGX groups (*p* < 0.01).

Surgical and chemical castration caused significantly higher OcS/BS for ORX, ORX + TES, DGX and DGX + TES groups compared to the baseline group (*p* < 0.05). The OcS/BS of ORX, ORX + TES and DGX groups was also significantly higher than the Sham group (*p* < 0.05). Supplementations with 50 and 100 mg/kg of quassinoid-rich EL extract to orchidectomised rats (ORX + EL50 and ORX + EL100 groups) resulted in lower OcS/BS compared to ORX group (*p* < 0.01). The OcS/BS value was also lower in ORX + EL25 group but the difference was not statistically significant (*p* = 0.053). The ORX + EL50 group also had a significantly lower OcS/BS compared to the ORX + TES group (*p* < 0.01). In degarelix-induced groups, only supplementations with 25 and 50 mg/kg of quassinoid-rich EL extract (DGX + EL25 and DGX + EL50 groups) resulted in significantly lower OcS/BS compared to the DGX group (*p* < 0.05).

The OS/BS of Sham, ORX + TES, ORX + EL25, ORX + EL50, ORX + EL100 and DGX + TES groups was significantly higher than the baseline group (*p* < 0.05). The OV/BV for all groups, except for DGX group was significantly higher than baseline group (*p* < 005). No significant differences were seen for ES/BS parameter (*p* > 0.05) (Table 3).

### 3.6. Structural Histomorphometric Parameters

BV/TV and TbN of ORX group were significantly lower, while TbSp was significantly higher than the baseline and Sham groups (*p* < 0.05). Similar patterns of BV/TV, TbN and TbSp were seen with the DGX group compared to the baseline and Sham groups (*p* < 0.01). Testosterone treatments significantly increased BV/TV and TbN and significantly reduced TbSp of ORX + TES and DGX + TES groups compared to their respective negative control groups (*p* < 0.05). The exception was for the TbN of ORX + TES group, which was not significantly different to the ORX group (*p* > 0.05). In general, supplementations of quassinoid-rich EL at all doses to degarelix groups could cause improvements in BV/TV, TbN, and TbSp parameters compared to the DGX group (*p* < 0.05). The BV/TV was also higher in the DGX + EL50 group compared to the DGX group but the difference was not statistically significant (*p* = 0.051). As for the orchidectomised group, only supplementation with 100 mg/kg quassinoid-rich EL extract (ORX + EL100) showed improvement in the TbSp compared to ORX group (*p* < 0.05). The TbN of the ORX + EL100 group was higher than the ORX group but the change was not significant (*p* = 0.059). No significant findings were found for the TbTh parameter (*p* > 0.05) (Table 4).

### 3.7. Dynamic Histomorphometric Parameters

ORX and DGX groups had significantly higher sLS/BS and significantly lower dLS/BS and MAR compared to Sham group (*p* < 0.01). Testosterone treatment to orchidectomised rats (ORX + TES group) could significantly reverse all the orchdectomy-induced changes on sLS/BS, dLS/BS and MAR parameters (*p* < 0.05). While, similar changes could only be seen on dLS/BS for DGX + TES group when compared to DGX group (*p* < 0.01). Quassinoid-rich EL extract supplementation at all doses significantly improved dLS/BS for both orchidectomised and degarelix-induced groups compared to their respective negative control groups (*p* < 0.05). Quassinoid-rich EL extract supplementation at all doses could improve sLS/BS of degarelix-induced groups but significant changes were only seen in orchidectomised rats supplemented with 25 and 100 mg/kg EL extract (*p* < 0.05). Similar effects were not seen for MAR parameter. MAR was significantly higher in ORX + EL50, ORX + EL100 and DGX + EL25 groups compared to their negative control groups (*p* < 0.05). MAR was also higher in DGX + EL50 but the difference was not significant (*p* = 0.057). There were also no significant differences noted for MS/BS and BFR/BS parameters (*p* > 0.05) (Table 5).

## 4. Discussion

In addition to pharmacological agents, nutrient supplements and functional foods have been considered as therapeutic options for male osteoporosis [38]. The use of medicinal plants in the prevention and treatment of osteoporosis is also gaining more interest as they may provide cost effective and convenient alternatives with fewer side effects. In this study, standardized quassinoid-rich EL extract was evaluated as an alternative option for the prevention of degenerative bone changes in osteoporosis due to androgen deficiency in rats.

In the present study, it was found that testosterone deficiency due to orchidectomy or chemical castration by degarelix resulted in unfavorable changes of static, structural, and dynamic bone histomorphometry indices. The deterioration of trabecular bone was evidenced by significant reductions in structural parameters such as bone volume (BV/TV) and trabecular number (TbN) as well as an increase in trabecular separation (TbSp). In an earlier study, only bone volume parameter was adversely affected by orchidectomy and supplementation with crude EL extract at 15 mg/kg dose was unable to reduce the bone volume changes [29]. In the present study, testosterone treatment was able to reduce degenerative bone changes by improving BV/TV, TbN and TbSp. Similar bone protective effects were also seen with supplementation of quassinoid-rich EL extracts to androgen-deficient rats, more in the case of degarelix-induced rats than orchidectomised rats. Based on the findings, quassinoid-rich EL was as effective as testosterone in protection against androgen deficient-bone structural changes, especially for the chemical castration model.

Static bone histomorphometry is used to quantitatively evaluate the activity of bone cells at a specific time [39]. It measures the number of osteoblasts and osteoclasts. In our present study, most of the findings were focused on the ObS/BS and OcS/BS of static histomorphometric parameters. Orchidectomy and degarelix administration caused more significant effects on the osteoclasts surface (OcS/BS) than the osteoblast surface (ObS/BS). The protective effects of testosterone and quassinoid-rich EL supplementation were demonstrated more on OcS/BS. The best doses of quassinoid-rich EL were 50 and 100 mg/kg for orchidectomised rats and 25 and 50 mg/kg for degarelix rats. Surprisingly, 50 mg/kg dose of quassinoid-rich EL was able to promote Obs/BS until it was higher than the Sham value. Based on the static histomorphometric parameters, quassinoid-rich EL offered bone protection in both orchidectomised and degarelix models with the 50 mg/kg dose demonstrating good activities.

Dynamic histomorphometry evaluation provides a quantitative assessment of bone formation over a period of time [34]. The results of dynamic histomorphometric analyses showed that the single-labeled surface (sLS/BS) was significantly increased, while the double-labeled surface (dLS/BS) and mineral apposition rate (MAR) were significantly decreased in both orchidectomised and degarelix-induced groups. The dLS/BS and MAR parameters correspond to new bone formation and bone surfaces with mineralization while the sLS/BS corresponds to bone surfaces with poor or no new bone formation [40]. The increase in sLS/BS with testosterone deficiency in this study indicated that either bone resorption activity was increased or bone formation was inadequate [41]. The sLS/BS was reduced by testosterone treatment and quassinoid-rich EL extract supplementation while dLS/BS was significantly improved in all treatment groups. These results showed that both testosterone treatment and quassinoid-rich EL extract supplementation were capable of increasing bone formation and reducing bone resorption. 

Although the dynamic histomorphometry parameters clearly showed that both testosterone treatment and quassinoid-rich EL extract supplementation were able to improve bone formation and bone resorption activities, the findings were not replicated with serum biochemical markers. Serum osteocalcin level was elevated in both orchidectomised and degarelix-induced groups. According to Erben, Eberle [6], bone loss may result in high bone turnover as shown by increased serum osteocalcin. Supplementations of quassinoid-rich EL extract to both orchidectomised and degarelix-induced groups also resulted in higher serum osteocalcin levels. Elevated bone turnover might suggest ongoing bone loss or may be as a result of active bone accrual [42]. Therefore, we speculated that the substantial increase in serum osteocalcin in the orchidectomised and degarelix-induced groups supplemented with quassinoid-rich EL extract was not due to bone loss but due to active bone formation instead. A significantly lower bone resorption marker, CTX was seen in all treated groups at the end of 10 weeks of study period compared to the baseline group. A previous study reported that serum CTX level was significantly lower at the end of the treatment period for all groups treated with tocotrienol, a vitamin E [43]. The anti-osteoporotic effects of EL were better illustrated in the dynamic histomorphometry parameters since findings in bone were more stable and were not affected by circadian rhythm compared to serum findings.

The failure of the testis to produce physiological levels of androgens particularly testosterone in hypogonadal men occur due to the disruption of one or more levels of the HPG axis [17]. As androgens play a significant role in the skeletal system, they protect men against osteoporosis. Orchidectomy was reported to cause 80% reduction in serum testosterone in male rats [6]. The testosterone levels of the orchidectomised and degarelix-induced rats in our study were significantly reduced and may be responsible for the changes in all bone parameters measured. As expected, testosterone treatment to both orchidectomised and degarelix-induced rats significantly raised the testosterone level. The increase in testosterone level in orchidectomised rats treated with testosterone was even higher than the Sham and baseline level. The testosterone levels in orchidectomised and degarelix-induced rats supplemented with quassinoid-rich EL extracts was significantly lower when compared to the Sham and baseline level, but interestingly, the marginal increase in testosterone level was also accompanied by an improvement in bone histomorphometric parameters in both orchidectomised and degarelix models.

Based on their roles, the relative balance of OPG and RANKL has been proposed to be an important determinant of bone resorption [44]. Androgens might maintain bone mass in part by increasing OPG and/or by suppressing RANKL [45]. A recent study demonstrated that androgen ablation via orchidectomy was associated with an increase in skeletal RANKL mRNA expression [46]. In the current study, though insignificant, there was an increase in the serum RANKL in the orchidectomised and degarelix-induced rats. The changes in peripheral serum RANKL were also not significant in an earlier study, but the RANKL levels in bone marrow plasma and bone marrow cells were found to be significantly increase in the orchidectomised rats. Nevertheless, there were significant reductions in serum OPG levels for both orchidectomised and degarelix-induced groups in this study and there were trends toward elevated serum OPG levels in the quassinoid-rich EL-supplemented groups. There were also trends towards suppression of serum RANKL seen in the testosterone group and quassinoid-rich EL-supplemented groups. A previous study reported that OPG-gene expression was up regulated with the supplementation of EL extract in the tibial bones of the orchidectomised rats but the changes in bone RANKL gene expression were not significant. It was postulated that, this might be an additional mechanism of EL in protecting against bone resorption induced by androgen deficiency.

To the best of our knowledge, this was the first study on the effects of standardized quassinoid-rich EL extract in male osteoporotic rat models, using both surgical and chemical castration. In this study, supplementation of quassinoid-rich EL extract did not show a dose-dependent effect on bone. In general, the skeletal improvement on histomorphometric indices for orchidectomised and degarelix-induced rats that received 25 and 50 mg/kg of quassinoid-rich EL extract was almost similar. Therefore, these two doses may be the best doses to offer bone protection against androgen deficiency. In fertility and reproductive studies, the effects of standardized quassinoid-rich EL extract on spermatogenesis were found to be optimum at 25 and 50 mg/kg [32]. However, quassinoid-rich EL extracts at higher dose of 100 mg/kg did not seem to worsen the bone parameters. In fact, some of the parameters were improved with this high dose. Moreover, standardized quassinoid-rich EL extract was reported to be non-teratogenic to fetal morphology, viscera and skeleton at 100 mg/kg. The doses of 100 mg/kg and below were considered safe for further clinical studies [47].

The marginal increase of testosterone level might contribute to one of the possible mechanisms by which EL extract supplementation reduced skeletal deterioration in this study. Since the deterioration in bone parameters was observed as an adverse effect of testosterone deficiency, a tiny increase of testosterone level by EL extract supplementation might be at least beneficial in reducing further changes to the bones in gonadal hormone-deficient male rats. Studies have demonstrated that circulating androgens and estrogens are protective of bone. In culture media, testosterone and 5α-dihydrotestosterone (5α-DHT) have been shown to promote proliferative and differentiative activities of osteoblast-like cells [48]. An in vitro study by Thu et al. (2017) showed that the standardized quassinoid rich EL extract has the potential to promote the proliferation and differentiation of osteoblast [49]. The proliferative and osteogenic effects of EL extract were compared with the positive control group that was treated with 5α-DHT. An earlier study reported that the ability of EL to promote proliferation in bone forming cells was slightly less than 5α-DHT while better efficacy was seen in promoting cell differentiation [50]. This may explain the beneficial effect of this extract on bone in vivo.

In conclusion, based on the histomorphometric indices, standardized quassinoid-rich EL extract can be considered to be as effective as testosterone in reducing bone degenerative changes in androgen-deficient osteoporosis models. This was partially achieved with a marginal increase in serum testosterone level compared to the estimated three-fold increase following testosterone treatment. Further investigation is warranted to clarify the exact mechanism by which quassinoid-rich EL extract exerted its anti-osteoporotic effects.

## Figures and Tables

**Table 1:** Bone histomorphometry parameters.

Parameters Measured	Abbreviation
**Structural Parameters**	
Trabecular Volume	BV/TV
Trabecular Thickness	TbTh
Trabecular Number	TbN
Trabecular Separation	TbSp
**Static Parameters**	
Osteoblast Surface	ObS/BS
Osteoclast Surface	OcS/BS
Eroded Surface	ES/BS
Osteoid Volume	OV/BV
Osteoid Surface	OS/BS
**Dynamic Parameters**	
Single-labeled Surface	sLS/BS
Double-labeled Surface	dLS/BS
Mineralizing Surface	MS/BS
Mineral Apposition Rate	MAR
Bone Formation Rate	BFR/BS

**Table 2:** Results of serum testosterone level.

	Mean (ng/mL)	SEM
Baseline	12.803	2.12
Sham	6.033 *	0.74
ORX	0.604 *^,a^	0.06
ORX + TES	19.263 *^,a,b^	2.018
ORX + EL25	1.132 *^,a,b,c^	0.03
ORX + EL50	1.042 *^,a,c^	0.21
ORX + EL100	1.186 *^,a,c^	0.083
DGX	0.594 *^,a^	0.02
DGX + TES	23.218 *^,a,d^	1.21
DGX + EL25	1.304 *^,a,e^	0.12
DGX + EL50	1.589 *^,a,d,e^	0.14
DGX + EL100	1.1314 *^,a,d,e^	0.03

Abbreviations: orchidectomised = ORX; TES = testosterone; DGX = degarelix-induced; EL = *Eurycoma longifolia*. Experimental groups: Sham—Sham-operated; ORX—orchidectomised control; ORX + TES—orchidectomised + testosterone injection; ORX + EL25—orchidectomised + 25 mg/kg EL extract; ORX + EL50—orchidectomised + 50 mg/kg EL extract; ORX + EL100—orchidectomised + 100 mg/kg EL extract; DGX—degarelix-induced; DGX + TES—degarelix-induced + testosterone injection; DGX + EL25—degarelix-induced + 25 mg/kg EL extract; DGX + EL50—degarelix-induced + 50 mg/kg EL extract; DGX + EL100—degarelix-induced + 100 mg/kg EL extract. Value expressed as mean ± SEM. *n* = 8 rats in each group. *p* < 0.05 is considered as significant. * Significant difference versus Baseline; ^a^ Significant difference versus Sham; ^b^ Significant difference versus ORX; ^c^ Significant difference versus ORX + TES; ^d^ Significant difference versus DGX; ^e^ Significant difference versus DGX + TES.

**Table 3:** Results of bone static histomorphometric parameters.

	ObS/BS (%)	OcS/BS (%)	ES/BS (%)	OS/BS (%)	OV/BV (%)
Baseline	8.77 ± 0.52	1.52 ± 0.36	5.43 ± 0.50	2.68 ± 0.39	3.07 ± 0.32
Sham	12.53 ± 0.75	2.96 ± 0.36	4.98 ± 0.57	5.02 ± 0.32 *	6.12 ± 0.41 *
ORX	9.74 ± 0.81	7.24 ± 0.44 *^,a^	4.13 ± 0.54	4.73 ± 0.31	5.94 ± 0.59 *
ORX + TES	11.17 ± 0.34	5.23 ± 0.24 *^,a^	4.81 ± 0.39	4.81 ± 0.57 *	6.27 ± 0.78 *
ORX + EL25	12.93 ± 0.77	4.11 ± 0.59	5.04 ± 0.47	5.74 ± 0.47 *	7.37 ± 0.62 *
ORX + EL50	15.72 ± 1.87 *	2.87 ± 0.28 ^b,c^	3.70 ± 0.39	5.81 ± 0.69 *	6.97 ± 0.66 *
ORX + EL100	12.40 ± 0.89	3.49 ± 0.42 ^b^	4.55 ± 0.41	5.63 ± 0.45 *	6.90 ± 0.82 *
DGX	12.16 ± 0.51	4.67 ± 0.23 *^,a^	5.98 ± 0.81	4.60 ± 0.21	5.32 ± 0.40
DGX + TES	19.36 ± 1.20 *^,a,d^	3.98 ± 0.35 *	4.42 ± 0.59	5.58 ± 0.30 *	7.75 ± 0.64 *
DGX + EL25	17.95 ± 0.94 *^,a,d^	2.38 ± 0.20 ^d^	4.76 ± 0.51	4.55 ± 0.27	6.12 ± 0.41 *
DGX + EL50	16.71 ± 1.45 *	2.55 ± 0.32 ^d^	3.63 ± 0.53	4.56 ± 0.59	5.93 ± 0.55 *
DGX + EL100	15.46 ± 1.19 *	2.91 ± 0.40	3.54 ± 0.40	4.59 ± 0.48	5.84 ± 0.40 *

Abbreviations: orchidectomised = ORX; TES = testosterone; DGX = degarelix-induced; EL = *Eurycoma longifolia*; ObS/BS = osteoblast surface; OcS/BS = osteoclast surface; ES/BS = eroded surface; OS/BS = osteoid surface; OV/BV = osteoid volume. Experimental groups: Sham, Sham-operated; ORX, orchidectomised control; ORX + TES, orchidectomised + testosterone injection; ORX + EL25, orchidectomised + 25 mg/kg EL extract; ORX + EL50, orchidectomised + 50 mg/kg EL extract; ORX + EL100, orchidectomised + 100 mg/kg EL extract; DGX, degarelix-induced; DGX + TES, degarelix-induced + testosterone injection; DGX + EL25, degarelix-induced + 25 mg/kg EL extract; DGX + EL50, degarelix-induced + 50 mg/kg EL extract; DGX + EL100, degarelix-induced + 100 mg/kg EL extract. Value expressed as mean ± SEM. *n* = 8 rats in each group. *p* < 0.05 is considered as significant. * Significant difference versus Baseline; ^a^ Significant difference versus Sham; ^b^ Significant difference versus ORX; ^c^ Significant difference versus ORX + TES; ^d^ Significant difference versus DGX.

**Table 4:** Results of bone structural histomorphometric parameters.

	BV/TV	TbTh	TbN	TbSp
Baseline	42.06 ± 2.06	139.86 ± 8.68	0.0030 ± 0.0001	193.62 ± 9.13
Sham	38.29 ± 2.90	167.09 ± 11.80	0.0029 ± 0.0002	282.06 ± 16.77
ORX	24.45 ± 0.73 *^,a^	114.37 ± 6.91	0.0021 ± 0.0001 *^,a^	377.25 ± 9.78 *^,a^
ORX + TES	38.63 ± 2.07 ^b^	149.65 ± 13.99	0.0026 ± 0.0002	237.97 ± 17.89 ^b^
ORX + EL25	31.55 ± 1.59 *	123.32 ± 3.71	0.0025 ± 0.0002	276.44 ± 25.99
ORX + EL50	28.96 ± 1.29 *	136.82 ± 7.62	0.0020 ± 0.0001 *^,a^	317.14 ± 16.59 *
ORX + EL100	32.36 ± 1.75 *	128.18 ± 5.83	0.0028 ± 0.0001	281.18 ± 15.56 ^b^
DGX	19.53 ± 0.94 *^,a^	98.96 ± 5.72	0.0018 ± 0.0001 *^,a^	419.25 ± 16.67 *^,a^
DGX + TES	33.08 ± 1.88 *^,d^	132.31 ± 20.49	0.0029 ± 0.0002 ^d^	212.10 ± 8.55 ^d^
DGX + EL25	30.01 ± 1.83 *^,d^	125.96 ± 13.59	0.0025 ± 0.0002 ^d^	243.18 ± 11.13 ^d^
DGX + EL50	29.76 ± 0.78 *	100.47 ± 7.44	0.0029 ± 0.0001 ^d^	240.97 ± 12.53 ^d^
DGX + EL100	33.56 ± 3.00 ^d^	107.95 ± 9.24	0.0029 ± 0.0002 ^d^	228.23 ± 10.78 ^d^

Abbreviations: orchidectomised = ORX; TES = testosterone; DGX = degarelix-induced; EL = *Eurycoma longifolia*; BV/TV = trabecular volume; TbTh = trabecular thickness; TbN = trabecular number; TbSp = trabecular separation. Experimental groups: Sham, Sham-operated; ORX, orchidectomised control; ORX + TES, orchidectomised + testosterone injection; ORX + EL25, orchidectomised + 25 mg/kg EL extract; ORX + EL50, orchidectomised + 50 mg/kg EL extract; ORX + EL100, orchidectomised + 100 mg/kg EL extract; DGX, degarelix-induced; DGX + TES, degarelix-induced + injection; DGX + EL25, degarelix-induced + 25 mg/kg EL extract; DGX + EL50, degarelix-induced + 50 mg/kg EL extract; DGX + EL100, degarelix-induced + 100 mg/kg EL extract. Value expressed as mean ± SEM. *n* = 8 rats in each group. *p* < 0.05 is considered as significant. * Significant difference versus baseline; ^a^ Significant difference versus Sham; ^b^ Significant difference versus ORX; ^d^ Significant difference versus DGX.

**Table 5:** Results of bone dynamic histomorphometric parameters.

	sLS/BS	dLS/BS	MAR	MS/BS	BFR/BS
Baseline	8.75 ± 0.68	9.50 ± 0.41	1.39 ± 0.09	40.29 ± 3.67	54.0 ± 7.55
Sham	6.29 ± 0.93	15.11 ± 1.31 *	1.35 ± 0.06	67.95 ± 9.06	73.39 ± 12.85
ORX	15.71 ± 1.21 *^,a^	6.33 ± 0.84 ^a^	0.76 ± 0.03 *^,a^	37.92 ± 5.74	40.59 ± 7.99
ORX + TES	8.18 ± 0.65 ^b^	10.99 ± 0.99 ^b^	1.28 ± 0.05 ^b^	48.23 ± 4.95	58.99 ± 8.45
ORX + EL25	6.94 ± 0.68 ^b^	13.80 ± 0.77 ^b^	1.05 ± 0.09	49.22 ± 9.33	55.35 ± 10.84
ORX + EL50	8.43 ± 1.24	11.95 ± 0.57 ^b^	1.33 ± 0.10 ^b^	49.47 ± 9.77	57.0 ± 16.27
ORX + EL100	6.56 ± 0.44 ^b^	13.07 ± 0.89 ^b^	1.23 ± 0.10 ^b^	42.31 ± 4.56	51.37 ± 6.93
DGX	16.94 ± 1.04 *^,a^	6.66 ± 0.73 ^a^	0.90 ± 0.05 *^,a^	51.12 ± 0.77	52.76 ± 3.72
DGX + TES	10.75 ± 1.66	13.01 ± 0.93 ^d^	1.26 ± 0.13	54.59 ± 7.85	68.37 ± 9.26
DGX + EL25	9.64 ± 0.93 ^c^	11.83 ± 0.89 ^d^	1.35 ± 0.09 ^d^	51.04 ± 5.61	69.20 ± 7.91
DGX + EL50	7.75 ± 0.83 ^c^	11.11 ± 1.04 ^d^	1.32 ± 0.09	51.59 ± 4.36	62.94 ± 13.70
DGX + EL100	6.22 ± 0.79 ^c^	11.67 ± 0.79 ^d^	1.22 ± 0.08	50.28 ± 6.83	59.28 ± 14.34

Abbreviations: orchidectomised = ORX; TES = testosterone; DGX = degarelix-induced; EL = *Eurycoma longifolia*; sLS/BS = single-labeled surface; dLS/BS = double-labeled surface; MS/BS = mineralizing surface; MAR = mineral apposition rate; BFR/BS = bone formation rate. Experimental groups: Sham, Sham-operated; ORX, orchidectomised control; ORX + TES, orchidectomised + testosterone injection; ORX + EL25, orchidectomised + 25 mg/kg EL extract; ORX + EL50, orchidectomised + 50 mg/kg EL extract; ORX + EL100, orchidectomised + 100 mg/kg EL extract; DGX, degarelix-induced; DGX + TES, degarelix-induced + testosterone injection; DGX + EL25, degarelix-induced + 25 mg/kg EL extract; DGX + EL50, degarelix-induced + 50 mg/kg EL extract; DGX + EL100, degarelix-induced + 100 mg/kg EL extract. Value expressed as mean ± SEM. *n* = 8 rats in each group. *p* < 0.05 is considered as significant. * Significant difference versus baseline; ^a^ Significant difference versus Sham; ^b^ Significant difference versus ORX; ^c^ Significant difference versus ORX + TES; ^d^ Significant difference versus DGX.
